# Preservation of Cognitive Performance with Age during Exertional Heat Stress under Low and High Air Velocity

**DOI:** 10.1155/2015/619103

**Published:** 2015-03-22

**Authors:** Heather E. Wright Beatty, Jocelyn M. Keillor, Stephen G. Hardcastle, Pierre Boulay, Glen P. Kenny

**Affiliations:** ^1^Human and Environmental Physiology Research Unit, School of Human Kinetics, Faculty of Health Sciences, University of Ottawa, 125 University Private, Montpetit Hall, Room 367, Ottawa, ON, Canada K1N 6N5; ^2^Flight Research Lab, National Research Council Canada, Ottawa, ON, Canada K1A 0R6; ^3^CanmetMINING Natural Resources Canada, Sudbury, ON, Canada P3E 5P5; ^4^Faculty of Physical Education and Sports, University of Sherbrooke, Sherbrooke, QC, Canada J1K 2R1

## Abstract

Older adults may be at greater risk for occupational injuries given their reduced capacity to dissipate heat, leading to greater thermal strain and potentially cognitive decrements. *Purpose.* To examine the effects of age and increased air velocity, during exercise in humid heat, on information processing and attention. *Methods.* Nine young (24 ± 1 years) and 9 older (59 ± 1 years) males cycled 4 × 15 min (separated by 15 min rest) at a fixed rate of heat production (400 W) in humid heat (35°C, 60% relative humidity) under 0.5 (low) and 3.0 (high) m·s^−1^ air velocity wearing coveralls. At rest, immediately following exercise (end exercise), and after the final recovery, participants performed an abbreviated paced auditory serial addition task (PASAT, 2 sec pace). *Results.* PASAT numbers of correct responses at end exercise were similar for young (low = 49 ± 3; high = 51 ± 3) and older (low = 46 ± 5; high = 47 ± 4) males and across air velocity conditions, and when scored relative to age norms. Psychological sweating, or an increased sweat rate with the administration of the PASAT, was observed in both age groups in the high condition. *Conclusion.* No significant decrements in attention and speeded information processing were observed, with age or altered air velocity, following intermittent exercise in humid heat.

## 1. Introduction

Performing physically demanding work in hot environments and/or while wearing protective clothing increases thermal strain, which has been associated with decrements in cognitive function, such as reduced short-term memory, recall, and discrimination [1]; reaction time, reasoning, and vigilance [2]; and arithmetic ability [3]. In some cases, however, improved cognitive performance has been observed during short duration continuous aerobic exercise (i.e., 60% VO_2peak_, 10 min duration; choice-discrimination) [4], during intense exercise (i.e., at/above anaerobic threshold; short-term memory) [5], and after bouts of continuous exercise of 20 and 40 min in duration (i.e., 70% VO_2peak_; mathematical computations) [6]. Attempts to reconcile these conflicting reports in the literature have proposed that they are due to variances in the participant groups (e.g., hydration status, level of heat acclimatization, and degree of sleep deprivation) and/or in the methodology (e.g., environmental conditions, mode of exercise, and type of difficulty of cognitive test) between studies. Although cognitive performance has been examined during and following exercise in the heat, the analysis is typically done using either change in hydration or exercise rate (e.g., exercise at a % of VO_2peak_, absolute work rate, or completion of predetermined work tasks) [1, 2, 7] as the independent variables. In addition, the above studies were conducted in young, healthy, active individuals (some middle-aged participants in Heckler and Croce [6]). Thus, no study has examined cognitive performance during exercise in the heat as a function of age or following intermittent exercise at a constant rate of thermal drive, which matches the work load required in many occupational settings [8], where attention and vigilance are crucial for worker safety.

Age-related decrements in heat loss responses of sweating and skin blood flow are known to compromise the thermoregulatory efficacy of older adults [9–11]. Consequently, concerns have been raised regarding the safety of older workers in occupations which require physically demanding tasks to be performed in the heat and/or while wearing protective clothing and the need for attention/concentration in operating equipment (e.g., deep underground and surface mining, hydroelectric utilities, steel and chemical plants, and others). Age-related decrements in heat loss responses are not always reflected in measurements of local heat loss responses (e.g., sweat rate) and core temperature [11–13]. Given that health and safety heat stress guidelines are based on core temperature [14], it must be recognized that increases in thermal strain and thus potentially age-related differences in cognitive performance [15] may still be present in older adults. However, the extent to which age-related differences in thermoregulation impact cognitive performance (e.g., information processing and attention) is currently unknown, particularly under conditions that restrict evaporative heat loss (i.e., humid heat and insulative protective clothing) which can result in increases in thermal strain. Furthermore, in attempts to mitigate thermal strain in workers, ventilation systems delivering large, and in some instances thermally conditioned, air volumes have been utilized in deep underground mining [16]. Such ventilation systems are required to limit the increase in environmental temperature due to the various energy transformations and heat sources within a mine while also providing a flow to promote worker cooling. The supply of suitable ventilation has been shown effective in reducing thermal strain (i.e., core temperature) in lab studies during exercise in heat in active young adults (i.e., 25–30 years) while wearing minimal clothing [17–19]. Moreover, in a recent report, increased air velocity was shown to be effective during intermittent exercise in humid heat in young (i.e., ~25 years) and older (i.e., ~60 years) males while wearing work coveralls [13]. It remains unknown, however, whether ventilation-mediated reductions in thermal strain impact cognitive performance in young and older adults.

The primary purpose of this study was to examine the influence of aging and increased air velocity on attention and speeded information processing, following intermittent exercise in humid heat under low (0.5 m·s^−1^) and high (3.0 m·s^−1^) air velocity work conditions in young (24.1 ± 0.5 years) and older (59.4 ± 1.2 years) males. Given that cognitive stress and/or increased mental workload have been shown to increase sweating (termed psychological sweating), identifying the effects of tasks which require sustained attention and working memory on thermoregulatory and cardiovascular responses would be applicable to occupations which require concentration and attention under potentially stressful conditions [20–22]. Thus, the secondary purpose of the study was to examine the influence of increased attentional requirements and increased working memory loads on thermal and cardiovascular responses. Accordingly, thermal and cardiovascular responses, and performance on a test requiring attention and speeded information processing, were compared for young and older males following intermittent exercise in humid heat (35°C, 60% relative humidity [RH]) under low and high air velocity conditions. The low velocity may be considered similar to that typically employed in industrial settings, such as mining for general occupational hygiene related exposure control, whereas the higher air velocity (i.e., 3.0 m·s^−1^) would be supplied to mitigate heat stress in workers. To be consistent with the required absolute work rate in many occupational settings [8], including deep underground mining, and a moderate-to-heavy work rate as defined by the American Conference of Governmental Industrial Hygienists (ACGIH) [14], a fixed rate of heat production of 400 W was utilized during exercise to maintain an equal heat load and therefore a similar level of thermal strain between groups. It was hypothesized that increased air velocity would be less effective in reducing both thermal and cardiovascular strain, as well as any cognitive decrements, in the older males as compared with young males. It was also hypothesized that psychological sweating in both young and older males would be present as a result of increased attention and working memory demands but might not be as evident during the low air velocity condition compared to high air velocity condition due to increased skin wettedness as a result of a reduced capacity for the evaporation of sweat.

## 2. Materials and Methods

### 2.1. Participants

Upon receiving approval from the University of Ottawa Health Sciences and Science Research Ethics Board, 9 young and 9 older healthy, active, nonheat acclimated, nonsmoking males, matched for height, mass, and body surface area, were recruited for the study. None of the participants had a history of respiratory, metabolic, cardiovascular, and/or hypertension disease, or skin conditions, and none were on any medication related to these conditions. All participants were moderately educated (at minimum) individuals from the general population and university communities with a wide range of occupations (e.g., students, construction, government, and general labour employees). Some participants from each group had moderate levels of scientific background due to prior research participation. All participants were informed of the experimental procedures, associated risks, and discomforts prior to providing written consent. Participant characteristics are shown in Table 1.

### 2.2. Experimental Design

All participants completed one preliminary screening session and two experimental sessions. During the preliminary session, participants underwent a progressive incremental test on a semirecumbent bike (one minute stages with 20 W incremental increases) to determine maximal oxygen uptake (VO_2peak_) (AMETEK models S-3A/1 and CD 3A, resp., Applied Electrochemistry, AEI Technologies, Pittsburg, PA, USA). Participants cycled until they could no longer maintain the predetermined cadence or stopped due to volitional fatigue. During the VO_2peak_ exercise test, continuous electrocardiogram monitoring was performed in the older participants (age > 50 years) (Pulse Biomedical Inc., Norristown, PA, USA). Blood pressure was measured at every 2nd stage or every 2 min. Percent body fat was calculated using the Siri equation [23], following the measurement of body density by hydrostatic weighing.

The two experimental sessions followed a minimum of 2 weeks after the preliminary session and were separated by a minimum of 72 hours but performed at the same time of day. Prior to arriving at the laboratory, participants were instructed to eat a normal breakfast and drink water ad libitum, while refraining from caffeine and exposure to thermal stimuli for 12 and 24 hours prior to each session, respectively. Participants were also asked to refrain from alcohol and exercise for 24 hours prior to each session. Upon arrival at the laboratory, participants provided a urine sample, inserted the rectal temperature probe, sat for a 20-minute thermoneutral resting baseline, and were instrumented with a heart rate monitor and skin temperature sensors. Participants donned underwear (*I*_cl⁡_ =  ~0.05 clo insulation), long underwear (*I*_cl⁡_ =  ~0.19 clo), a t-shirt (*I*_cl⁡_ =  ~0.10 clo), work coveralls (*I*_cl⁡_ =  ~0.61 clo), socks (*I*_cl⁡_ =  ~0.04 clo), running shoes (*I*_cl⁡_ =  ~0.04 clo), work gloves, and a hard hat. Participants subsequently entered the thermal chamber (Can-Trol Environmental Systems Limited, Markham, ON, Canada) regulated at 35°C and 60% RH with an air velocity of 0.5 (low, first experimental session) or 3.0 (high, second experimental session) m·s^−1^ where they rested for 30 min while baseline measurements were obtained. They then performed four 15-minute bouts of cycling at a fixed rate of metabolic heat production of 400 W, a moderate-to-heavy intensity as defined by the ACGIH [14], separated by 15-minute rest periods with a final resting recovery of 30 min in duration. This rate of heat production is comparable to the absolute work rate required in occupational settings (e.g., mining) that are often performed in hot environments [8, 14].

### 2.3. Physiological and Cognitive Measurements

#### 2.3.1. Metabolic Heat Production

To establish a fixed rate of metabolic heat production of 400 W, the ergometer resistance was adjusted based on the concurrent measurements of expired oxygen and carbon dioxide concentrations (AMETEK models S-3A/1 and CD 3A, resp., Applied Electrochemistry, AEI Technologies, Pittsburg, PA, USA). Metabolic heat production was taken as the difference between metabolic energy expenditure and the resistance, in Watts, on the ergometer. Metabolic energy expenditure (*M*) was calculated from 30-second average values for oxygen uptake (VO_2_), according to the following equation.

(1) MWatts =V˙O2∗RER−0.7/0.3∗ec+1−RER/0.3∗ef60,

where RER is the respiratory exchange ratio (CO_2_ produced/O_2_ uptake), *e*_*c*_ is the caloric equivalent of a liter of oxygen when carbohydrates are oxidized (21.116 kJ), and *e*_*f*_ is the caloric equivalent of a liter of oxygen when fat is oxidized (19.606 kJ) [24]. Stable metabolic heat production values were typically attained within the first 5 min of the first exercise bout, following which minimal adjustments were made to maintain 400 W.

Absolute oxygen uptake was not different between the young (1358.24 ± 15.39 and 1357.11 ± 15.58 mL·min^−1^) and older (1363.05 ± 17.67 and 1344.21 ± 17.43 mL·min^−1^) males nor between air velocity conditions.

#### 2.3.2. Core Temperature

Rectal temperature (*T*_re_) was measured continuously using a pediatric thermocouple probe (Mon-a-therm Nasopharyngeal Temperature Probe, Mallinckrodt Medical, St. Louis, MO, USA), inserted 12 cm past the anal sphincter. Temperature data were collected using a HP Agilent data acquisition module (Model 3497A) at a sampling rate of 15 sec and simultaneously displayed and recorded on a personal computer with LabVIEW software (Version 7.0, National Instruments Corporation).

#### 2.3.3. Local Sweat Rate

Local sweat rate (LSR) was measured using a 3.8 cm^2^ ventilated capsule affixed to the skin on the forearm (LSR_FA_) and upper back (LSR_UB_) with an adhesive ring and topical skin glue (Collodion HV, Mavidon Medical, Lake Work, FL, USA). Anhydrous compressed air was passed through the capsules at a rate of 1 L·min^−1^. Water content of the effluent air was measured using high precision dew point mirrors (Model 473, RH systems, Albuquerque, NM, USA). Local sweat rate was determined by calculating the difference in water content between the effluent and influent air for each capsule, multiplied by the flow rate, and normalized for the skin surface area under the capsule.

#### 2.3.4. Heart Rate

Heart rate was measured continuously using a Polar coded transmitter and recorded with a Polar Advantage interface and Polar Precision Performance software (Polar Electro Oy, Finland).

#### 2.3.5. Cognitive Function Test

Due to the short work-to-rest cycles (15 min exercise-15 min rest; according to the ACGIH guidelines [14]), a portion of the Victoria Computerized Adaptation of the paced auditory serial addition task (PASAT; 2 sec pace, 3 min test, nonstandard administration) [25], as a measure of the capacity for sustained attention and speeded information processing, was administered to participants at resting baseline (baseline, after sitting in the warm/humid environment for 20 min), at the end of the 4th/final exercise bout (end exercise), and at the end of the 30 min final recovery (end recovery). The number of correct responses for each test was recorded and scores were normalized relative to age norms for the administered condition [26] as *Z* scores and percentiles. During the preliminary session, the 2-second pace segment of the PASAT was administered repeatedly, with breaks, until a stable score was obtained in order to counter any practice effects during the experimental sessions. The number of repetitions required to reach a stable score (defined as 2 unchanged scores in succession) averaged 4–6 repetitions for each group. In addition, the PASAT instructions were reviewed and practice administration of the 2-second pace was completed at the beginning of each experimental session.

### 2.4. Statistical Analyses

A one-way ANOVA was performed to compare age, height, mass, body surface area, % body fat, and VO_2peak_ of the young versus older males. A three-way analysis of variance (ANOVA) with 1 between grouping factor (young and older) and 2 repeated factors for condition (low and high) and time (baseline, end exercise, and end recovery) was performed on *T*_re_, heart rate, percent of maximum heart rate, LSR_FA_, LSR_UB_, and the PASAT scores (# of correct responses, *Z* scores, and percentiles). PASAT raw scores are reported, in addition to the normative values for the full administration of the test. To examine the effects of the PASAT administration on *T*_re_, heart rate, LSR_FA_, and LSR_UB_, a 2-way ANOVA with 1 between grouping factor (just prior to the start of the PASAT and the average of these measures over the 3-minute duration of the PASAT) and 1 repeated factor for time (baseline, end exercise, and end recovery) was performed on the *T*_re_, heart rate, LSR_FA_, and LSR_UB_ for the young and older males during the low and high air velocity conditions. When a significant *F*-ratio was obtained, a Newman-Keuls post hoc procedure was used to isolate differences. Significance was assumed for *P* ≤ 0.05 (trends were noted for 0.05 > *P* ≤ 0.10).

## 3. Results

### 3.1. Effects of Age and Air Velocity

**Figure 1:**
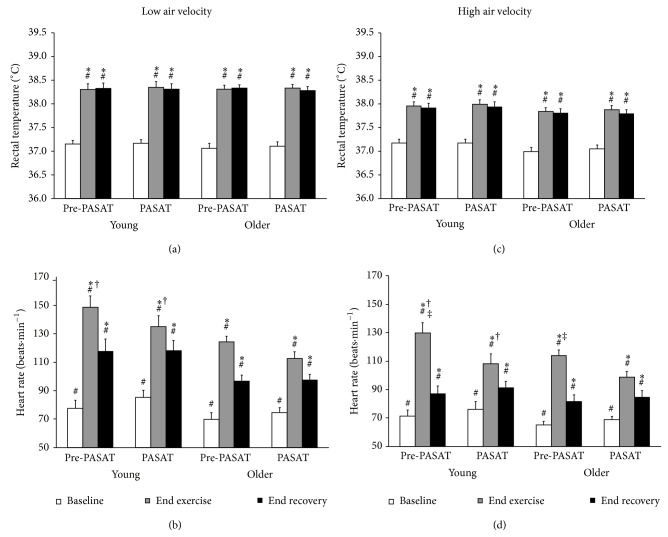
Rectal temperature ((a) and (c)) and heart rate ((b) and (d)) at the start of the paced auditory serial addition task (PASAT) and averaged over the 3-minute duration of the PASAT when administered at baseline (white), end of the 4th exercise bout (end exercise; grey), and end of the final recovery (end recovery; black) in young and older males under low and high air velocity. Values are mean ± SE. ∗ Main effect of time; end exercise and end recovery versus baseline for rectal temperature; end exercise and end recovery versus all time points for heart rate; # main effect of air velocity condition; † main effect of age; ‡ significant difference between pre-PASAT and PASAT within each air velocity condition and age group.

**Figure 2:**
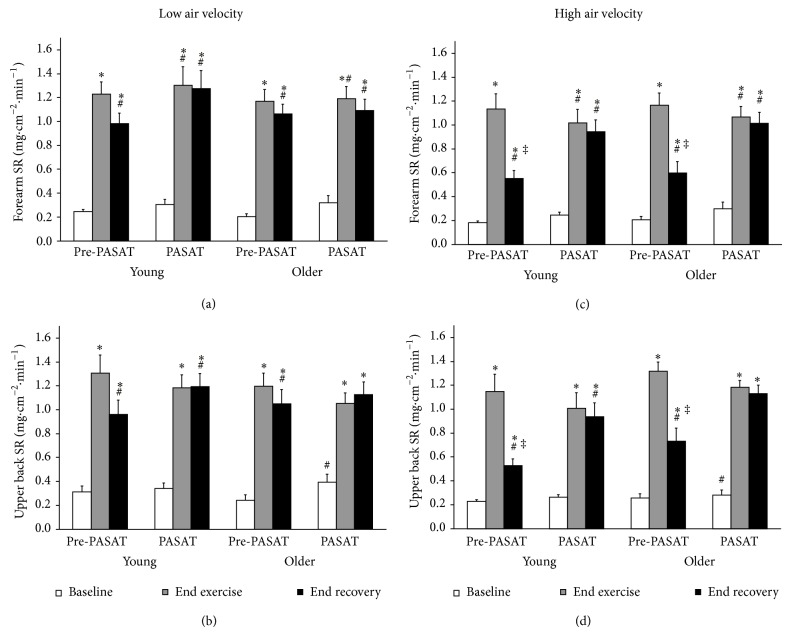
Local sweat rate (SR) at the forearm ((a) and (c)) and upper back ((b) and (d)) at the start of the paced auditory serial addition task (PASAT) and averaged over the 3-minute duration of the PASAT when administered at baseline (white), end of the 4th exercise bout (end exercise; grey), and end of the final recovery (end recovery; black) in young and older males under low and high air velocity. Values are mean ± SE. Notes: forearm *n* = 8 for older high condition; upper back *n* = 7 for young low condition, *n* = 8 for older low condition, and *n* = 8 for older high condition. ∗ Main effect of time; end exercise and end recovery versus all time points for pre-PASAT; end exercise and end recovery versus baseline for PASAT; # main effect of air velocity condition; ‡ significant difference between pre-PASAT and PASAT within each air velocity condition and age group.

**Figure 3:**
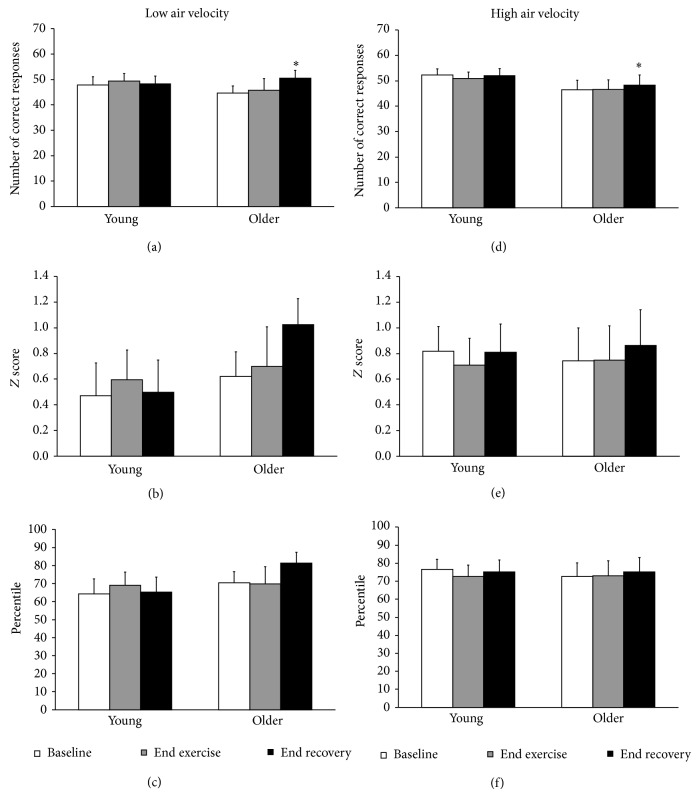
Paced auditory serial addition task # of correct responses ((a) and (d)); *Z* score relative to age norms ((b) and (e)); percentile relative to age norms ((c) and (f)) at baseline (white), end of the 4th exercise bout (end exercise; grey), and end of the final recovery (end recovery; black) in young and older males under low and high air velocity. Values are mean ± SE. ∗ Main effect of time; end recovery versus baseline and end exercise.

During the low and high air velocity conditions, no overall differences were observed between the young and older males for *T*_re_ (Figures 1(a) and 1(c)), LSR_FA_ (Figures 2(a) and 2(c)), LSR_UB_ (Figures 2(b) and 2(d)), PASAT *Z* score (Figures 3(b) and 3(e)), or PASAT percentile (Figures 3(c) and 3(f)). A group *x* time interaction was observed for heart rate, such that heart rate was greater in the young males compared to older males at end exercise with a trend at end recovery (*P* = 0.084) (Figures 1(b) and 1(d), trend for age main effect *P* = 0.067). There were no differences between the young and older males for percent of maximum heart rate in either the low condition at baseline (young = 41.6  ±  2.5, older = 44.1  ±  2.3%), end exercise (young = 80.0 ± 3.2, older = 77.3 ± 1.2%), or end recovery (young = 62.7 ± 3.3, older = 64.9 ± 2.0%), or the high condition at baseline (young = 39.0  ±  2.1, older = 41.1  ±  1.1%), end exercise (young = 67.9 ± 2.9, older = 71.1 ± 2.0%), or end recovery (young = 46.4 ± 2.9, older = 53.3 ± 2.3%). A group *x* time interaction was observed for PASAT number of correct responses, such that the number of correct responses were greater at end recovery compared to baseline and end exercise for the older males (Figures 3(a) and 3(d)).

Significant differences were observed between the low and high air velocity conditions for *T*_re_ (Figures 1(a) and 1(c)), heart rate (Figures 1(b) and 1(d)), percent of maximum heart rate, LSR_FA_ (Figures 2(a) and 2(c)), and LSR_UB_ (Figures 2(b) and 2(d)). A condition *x* time effect was observed for *T*_re_, heart rate, percent of maximum heart rate, LSR_FA_, and LSR_UB_, such that all time points were different from each other except for low versus high at baseline for *T*_re_, LSR_FA_, and LSR_UB_, and low versus high at end exercise for LSR_FA_ and LSR_UB_. No significant differences were observed between the low and high air velocity conditions for PASAT number of correct responses (Figures 3(a) and 3(d)), PASAT *Z* score (Figures 3(b) and 3(e), trend for condition main effect *P* = 0.076), or PASAT percentile (Figures 3(c) and 3(f), trend for condition main effect *P* = 0.063).

A significant effect of time was observed in the low and high air velocity conditions for *T*_re_ (baseline versus end exercise and end recovery), heart rate, percent of maximum heart rate, LSR_FA_, and LSR_UB_, (all time points different from each other), and PASAT number of correct responses (baseline and end exercise versus end recovery for older males) but not PASAT *Z* score (trend for time main effect *P* = 0.061) or PASAT percentile.

### 3.2. Effects of the PASAT Administration

The administration of the PASAT did not result in changes in *T*_re_; however, it resulted in a significant reduction in heart rate for the young and older males during the high air velocity condition at end exercise (trend for older during low at end exercise, *P* = 0.053). The PASAT also resulted in a significant increase in LSR_FA_ and LSR_UB_ for the young and older males at end recovery during the high air velocity condition.

## 4. Discussion

Cognitive function, specifically attention and capacity of information processing, was compared using an abbreviated version of the PASAT, in young and older males prior to and following intermittent exercise, performed at a fixed rate of heat production of 400 W. This rate of heat production is equivalent to a moderate-to-high workload according to the ACGIH guidelines [14]. The young and older males performed similarly on the PASAT (i.e., absolute number of correct responses and relative to age norms [26]) prior to and following intermittent exercise while wearing work coveralls in both the low and high air velocity conditions. This was paralleled by similar thermal (i.e., *T*_re_ and LSR at both skin sites) and cardiovascular (i.e., percent of maximum heart rate) responses between the age groups in both air velocity conditions. In addition, neither the young nor the older males demonstrated decrements in performance on the PASAT in either air velocity condition despite the high air velocity significantly reducing thermal (i.e., *T*_re_ and LSR at both skin sites) and cardiovascular (i.e., heart rate and percent of maximum heart rate) strain equally in both age groups. Psychological sweating was apparent in both the young and older males at the end of recovery in the high but not the low air velocity condition.

### 4.1. Effects of Age and Air Velocity on Cognitive Performance

While previous studies have reported decrements in cognitive performance, such as impaired short-term memory, recall, and vigilance, during exercise in the heat in young individuals [1–3], no study has examined potential age-related differences in cognitive function during work in humid heat. Reductions in the rate of information processing have been strongly linked to age-related changes in cognitive function [27]; however, young and older adults perform similarly on tests such as the PASAT that require overlearned arithmetic skills in conjunction with rapid information processing and sustained attention [28, 29]. It was hypothesized that when subjected to thermal stress the older males might demonstrate impairments in attention and speed of information processing on a test resistant to the effects of age-related cognitive decline (PASAT). However, the young and older males demonstrated a similar cognitive performance (i.e., attention and information processing) under heat stress during both the low and high air velocity conditions. Given that the population group used in the current study likely had different work experience, heat exposure backgrounds, and/or education levels as compared to some industry workers, it would be of great benefit to the occupational stakeholders if this study were repeated under field conditions and/or in the laboratory with industrial workers. Similar cognitive performance was observed between the age groups with the exception of a time effect for the older males, whereby the number of correct responses on the PASAT was significantly higher at end exercise and end recovery compared to baseline. Such an improvement in performance 30 min following exercise may be due to a practice effect over time which has previously been noted for this test [28]. The practice effects might be expected to be of a larger magnitude (despite repeated practice) due to the abbreviated quarter of the items available in the computer test being repeated at each session providing multiple exposure to a small subset of the items. It is perhaps not surprising that, given some practice, the older males were to benefit from these repeated exposures more than the young males as it has been hypothesized that generational differences in the overlearning of addition tasks may influence performance on this test [30]. Overall, the similar cognitive performance at the end of exercise between age groups was paralleled by similar thermal (i.e., *T*_re_ and LSR at both sites) and cardiovascular (i.e., percent of maximum heart rate) responses in both the young and older males within each of the air velocity conditions. The lack of a significant difference in cognitive performance following exertional heat stress is consistent with other studies reporting minimal or no changes in cognitive function when fluid replenishment was provided to avoid dehydration [31] or with dehydration levels of 2.6% or less body mass loss [32]. Alternatively, Szinnai et al. [32] suggested that when dehydration is developed slowly, as in the case with the current study over the duration of 2-3 hours, healthy individuals are better able to accommodate to the increased tiredness and reduced alertness (e.g., exercise arousal effect) which mitigates any reductions in the capacity of information processing mechanisms. In the current study, % body mass changes were similar between the young and older males following the low (2.4 ± 0.2 and 2.7 ± 0.3%, resp.) and high (1.8 ± 0.1 and 1.9 ± 0.2%, resp.) conditions, similar to or lower than the changes reported by Szinnai et al. [32]. This was despite the high air velocity being effective in reducing the level of dehydration in both age groups (detailed hydration status changes have been previously published [13]).

The increase in air velocity during the high condition was also effective in reducing thermal (i.e., *T*_re_, LSR at both skin sites) and cardiovascular (i.e., heart rate, percent of maximum heart rate) strain equally in both young and older males; however, the benefits of the high air velocity condition were not reflected in cognitive performance. Interestingly, Morley et al. [7] detected alterations in cognitive performance only at or beyond 1 hour after exercise, following 50 min of continuous treadmill exercise (i.e., walking 4.5 km·hr^−1^) in young, active adults (~28 years) wearing thermal protective clothing. These alterations in cognitive performance were at a high physiological stress where visceral temperatures were ~39.0°C and heart rates were ~170 bpm at the end of exercise. Thus, at the levels of thermal strain (i.e., *T*_re_ at end exercise of 38.0–38.3°C) and dehydration (i.e., 1.8–2.7% body mass loss) observed in the current study, young and older males were able to maintain attention during the conduct of speeded information processing without significant decrements in performance. This was despite anecdotal feedback from the participants in both groups that the PASAT was perceived as being more difficult during the low compared to high air velocity condition and the apparent presence of a practice effect for the older males. Decrements in cognitive performance, and potentially age-related differences, might have been observed with greater levels of dehydration (i.e., >3.0%), at greater levels of thermal strain (i.e., *T*_re_ ≥ 39.0°C), with longer duration intermittent exercise, and/or with the completion of a full cognitive test battery that manipulates task difficulty, as compared with the partial administration of the PASAT used in the current study.

### 4.2. Evidence of Psychological Sweating

Eccrine sweating is most commonly associated with exercise and exposure to heat, in which thermal stressors are driving the glandular responses; however, nonthermal stresses, such as mental stress, anxiety, fear, and pain, can also result in increased sweating, also known as psychological sweating [20, 22, 33]. Administration of the PASAT has been demonstrated to be a reliable method of inducing psychological stress in the laboratory [34]. Older studies suggested that psychological sweating is only observed on the skin of the hands and feet [35, 36] and can be inhibited by thermal stimulation when both thermal and psychological stimuli are present [35]. Conversely, more recent studies have shown a larger distribution of psychological sweating in thermoneutral individuals [22] and when stimulated by passive heating [33]. Novel to the current study was the observation of psychological sweating following exertional heat stress given that the local sweating at both the forearm and upper back skin sites increased significantly as a result of the PASAT administration in both young and older males 30 min following the final exercise bout in the high air velocity condition. Therefore, the current findings dispute previous research which suggested psychological sweating to be inhibited by thermal stimuli [35], and concurred with more recent research that both thermal and psychological sweating can occur simultaneously [33]. Despite the similar sweat rates measured at both skin sites at the end of exercise prior to the PASAT, between the low and high air velocity conditions, the effectiveness of evaporative cooling was evident in the high air velocity condition as reflected in the significantly reduced core temperatures in both young and older males. Thus, for occupations that require sustained attention, vigilance, and ongoing information processing during work in the heat, it may be plausible that increased psychological sweating may subject workers to an increased sweat loss and thus risk of dehydration, when the environment permits effective evaporative heat loss from sweating.

## 5. Conclusions

Young and older males demonstrated similar performance on a test of attention and speeded information processing, paralleled by similar thermal and cardiovascular responses, prior to and following intermittent exercise at a fixed rate of heat production (400 W, moderate-to-heavy work as defined by the ACGIH guidelines) while wearing standard work coveralls in humid heat (35°C, 60% RH) within each of the low (0.5 m·s^−1^) and high (3.0 m·s^−1^) air velocity conditions. Young and older males also demonstrated similar cognitive performance between the low and high air velocity conditions despite the high air velocity significantly reducing thermal (i.e., *T*_re_ and LSR at both skin sites) and cardiovascular (i.e., heart rate and percent of maximum heart rate) strain equally in both young and older males. The psychological sweating in the young and older males at the end of recovery in the high but not the low air velocity condition may be indicative of a greater risk for increased dehydration under conditions which permit effective evaporative heat loss from sweating. Thus, no significant decrements in attention and speeded information processing were observed, with age or altered air velocity, following intermittent exercise in humid heat.

## Figures and Tables

**Table 1:** Participant characteristics.

	Age	Height	Mass	BSA	Body fat	VO_2peak_
	(yr)	(cm)	(kg)	(m^2^)	(%)	(mLO_2_·kg^−1^·min^−1^)
Young	24.1 (0.5)^a^	174.6 (3.2)	79.4 (2.6)	1.95 (0.05)	15.5 (2.7)^a^	49.2 (2.6)^a^
Older	59.4 (1.2)	174.2 (1.2)	81.5 (4.1)	1.96 (0.05)	25.6 (2.6)	40.4 (2.6)

Note: values are mean (SE). Body surface area (BSA); maximal aerobic power (VO_2peak_).

^a^Significantly different than older males.
